# Local coalitions as an underutilized and understudied approach for promoting tobacco control in low- and middle-income countries

**DOI:** 10.7189/jogh.09.010301

**Published:** 2019-06

**Authors:** Carla J Berg

**Affiliations:** Department of Behavioral Sciences and Health Education, Rollins School of Public Health, Emory University, Atlanta, Georgia, USA

Cigarette smoking is the second leading risk factor for death worldwide [1]. Low- and middle-income countries (LMICs) are disproportionately affected by tobacco-related morbidity and mortality [1]. Because of the burden of tobacco use on global health, the World Health Organization developed the Framework Convention on Tobacco Control, which mandates the implementation of a range of evidence-based tobacco control policies [2].

**Figure 1:**
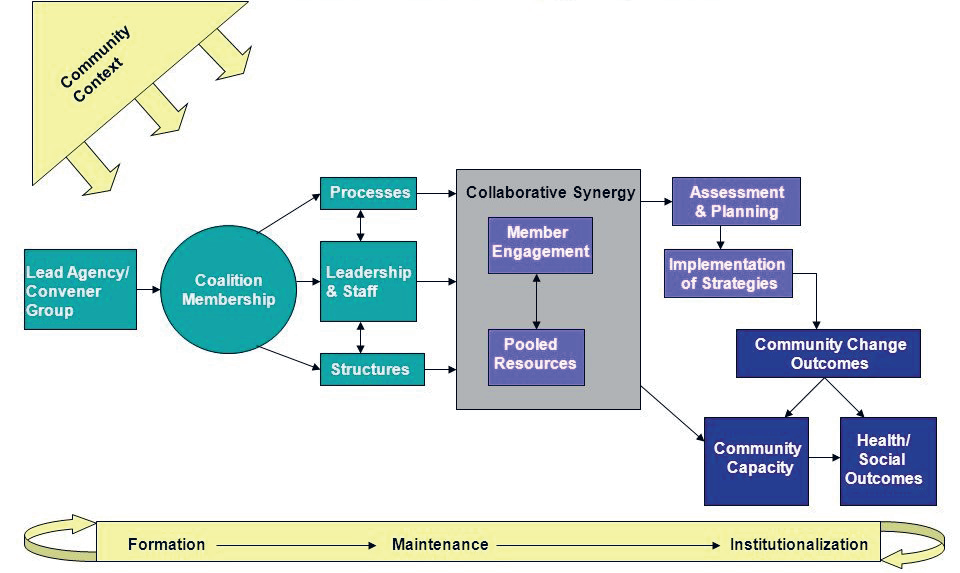
Community coalition action theory. Butterfoss & Kegler, 2009 [7].

Multi-sectoral local coalitions aligning civil society and local public health agencies have been effective in changing policy and have been particularly relevant to advancing tobacco control policies [3-6]. Community Coalition Action Theory (CCAT) synthesizes research on characteristics and processes of community coalitions (**Figure 1**) [7,8]. CCAT posits that coalitions form due to a threat, opportunity, or mandate. Local public health agencies often serve as the lead, with partners from a range of community sectors (eg, education, health care, non-profit agencies). Coalition members pool their individual and organizational resources (eg, connections/access, tangible resources, perspectives). Coalitions develop appropriately-tailored action plans and intervention strategies informed by best practices for policy change. Implementation of these strategies should result in community change, including improvements in policies, programs, and practices. These community changes, in turn, lead to health and social outcomes of interest (eg, reductions in secondhand smoke exposure). Community changes also lead to increases in community capacity to identify and address other issues.

**Figure:**
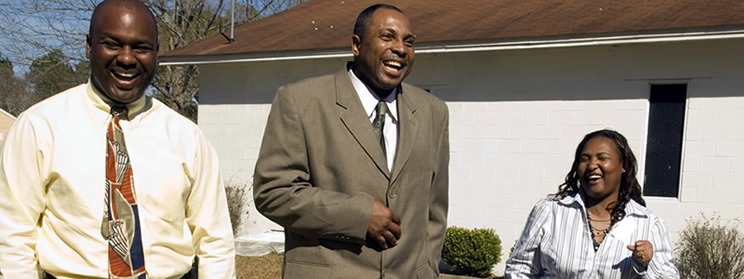
Photo: From Emory Prevention Research Center (used with permission)

This collaborative approach has been applied to tobacco broadly in high-income countries (HICs) but to a lesser degree in LMICs. Moreover, local coalitions may not be acceptable in political contexts with less democratic histories, such as former Soviet Union countries. Instead, policy change, including tobacco control policy change, has largely been initiated at the national level. While this is an efficient way to create change, compliance often remains poor [9]. Thus, parallel work may be required at the local level to build support for policies, shift social norms, and encourage compliance. Indeed, community coalitions are key in such processes [6]. Moreover, engaging civil society in tobacco control at the local level may be important, as they can play key roles such as advocates, watchdogs, and coalition builders [10].

The state of the research and practice indicates important opportunities to advance tobacco control, potentially ultimately curtailing the global burden of tobacco. Most critical, examining the feasibility, acceptability, and effectiveness of coalitions in LMICs and in contexts with different sociopolitical histories may be critical in advancing tobacco control in high-risk communities. Moreover, because LMICs have limited resources (eg, financial, human), it is crucial to understand coalition characteristics and processes and sociocontextual factors (eg, political context) that foster success in order to best leverage these resources.

In conclusion, future research is needed to answer fundamental questions regarding whether coalitions can significantly impact tobacco control in LMICs or those with less democratic histories, and, if so, how the CCAT and its constructs operate in these communities, providing a theoretical and empirical basis to inform future local tobacco control work. Ultimately, such research could catalyze future action-oriented science and evidence-based practice to support tobacco control progress globally.
